# Radiation Damage Mechanisms and Research Status of Radiation-Resistant Optical Fibers: A Review

**DOI:** 10.3390/s24103235

**Published:** 2024-05-20

**Authors:** Jicong Li, Qi Chen, Jia Zhou, Zhi Cao, Tianchi Li, Fang Liu, Zhongyuan Yang, Shangwen Chang, Keyuan Zhou, Yuzhou Ming, Taihong Yan, Weifang Zheng

**Affiliations:** China Institute of Atomic Energy, P.O. Box 275 (26), Beijing 102413, China; a2942257643@163.com (J.L.); larychen1995@163.com (Q.C.); 13521400205@163.com (J.Z.); caoz1020@163.com (Z.C.); liuxinyu741@sohu.com (F.L.); tracyiforever@163.com (Z.Y.); cswcsw1@sina.com (S.C.); keyuan3000@139.com (K.Z.); myz0228@163.com (Y.M.); yanthcn@163.com (T.Y.)

**Keywords:** radiation damage, radiation-resistant, optical fibers

## Abstract

In recent years, optical fibers have found extensive use in special environments, including high-energy radiation scenarios like nuclear explosion diagnostics and reactor monitoring. However, radiation exposure, such as X-rays, gamma rays, and neutrons, can compromise fiber safety and reliability. Consequently, researchers worldwide are focusing on radiation-resistant fiber optic technology. This paper examines optical fiber radiation damage mechanisms, encompassing ionization damage, displacement damage, and defect centers. It also surveys the current research on radiation-resistant fiber optic design, including doping and manufacturing process improvements. Ultimately, it summarizes the effectiveness of various approaches and forecasts the future of radiation-resistant optical fibers.

## 1. Introduction

As technologies like laser cutting [1,2,3,4] and fiber optic communication [5,6,7,8] rapidly evolve, optical fibers are seeing increasingly widespread applications across various fields. Beyond traditional communication, optical fibers have found extensive use in recent years in cutting-edge areas such as sensing [9,10,11], measurement [12,13], control [14,15], and data collection [16,17]. These applications extend to diverse environments, including high-energy radiation scenarios [18] like nuclear explosion diagnostics [19], internal monitoring of nuclear reactors [20,21,22], nuclear fuel reprocessing [23], disinfection of medical endoscopes [24,25], underwater fiber optic cable communication [26], and aerospace technology [27], among others.

Clearly, optical fibers are utilized not only in prolonged exposure to low-dose radiation environments but also in high-dose-rate, high-level radiation environments. In these scenarios, radiation such as X-rays, gamma rays, and neutron radiation can induce damage to the optical fibers, leading to a reduction in their capabilities and overall performance [28,29,30]. In severe instances, this radiation-induced damage can directly compromise the safety and reliability of the optical fibers [31]. Therefore, it is paramount to investigate the characteristics of optical fibers in various radiation environments, understand the mechanisms of defect formation, and diligently work towards improving and enhancing the radiation resistance of optical fibers.

This paper delves into the mechanisms of radiation-induced loss in optical fibers, encompassing ionization damage at the microscale, displacement damage, and macroscopic effects. Additionally, it surveys the current landscape of research on radiation-resistant fiber optic design, including methods such as doping for enhanced radiation resistance and advancements in manufacturing processes to bolster radiation resilience. Ultimately, it consolidates the efficacy of these methods in enhancing radiation resistance and offers insights into the future trajectory of radiation-resistant optical fibers.

## 2. Radiation Damage Mechanisms in Optical Fibers

### 2.1. Microscopic Effects of Irradiation Damage

The micro-level impact of radiation on optical fibers primarily arises from two phenomena: ionization damage and displacement damage, resulting in the formation of point defects. These radiation-induced defects are predominantly associated with the optical absorption (OA) bands. Consequently, they can induce structural modifications in both the core and cladding of the optical fiber, ultimately influencing its performance [32,33]. The damage inflicted on optical fibers by radiation predominantly manifests in two forms: displacement damage and ionization damage, arising from the energy exchange process between radiation and fibers [34,35]. Displacement damage stems primarily from high-energy particles, while ionization damage is primarily attributable to charged particles and electromagnetic waves. However, the energy threshold for displacement damage is usually significantly higher than that for ionization damage. Consequently, practical research tends to focus more on ionization damage.

#### 2.1.1. Ionization Damage

For optical fibers, ionization damage primarily involves the ionization of atomic nuclei by high-energy charged particles or electromagnetic waves, resulting in the creation of electron–hole pairs. Subsequently, due to the capture of electrons or holes by precursor defects, color center defects are generated. Charged particles, such as β particles and α particles, interact with outer atomic electrons via the Coulomb force, leading to the absorption of energy by the outer electrons and their transition to higher energy levels, a phenomenon known as excitation. If the energy absorbed by the outer electrons exceeds their binding energy, they become ionized, forming free-moving electrons [36]. Furthermore, the ionization process also generates secondary electrons, which, when their energy is sufficiently high, interact with other electrons, causing additional ionization damage [37].

Griscom et al. provided a comprehensive insight into the mechanism of self-trapped holes (STHs) in α-SiO_2_ [38]. They conducted analyses using electron spin resonance spectroscopy on various samples including high-OH flame hydrolytic, low-OH O_2_-plasma fused, sol–gel, and less pure isotopically enriched materials. Their findings revealed that under X-ray irradiation at 100 keV or excimer-laser illumination at 77 K and 6.4 eV, STHs could be considered as holes trapped on normal bridging oxygen in the α-SiO_2_ lattice when stabilized below ~180 K. However, when stabilized below ~140 K, STHs were perceived as holes trapped on two normal oxygens, akin to the VK center in alkali halides. Furthermore, Devine et al. elaborated on five potential defects serving as sources of positive fixed oxide charge.

#### 2.1.2. Displacement Damage

Displacement damage primarily occurs when high-energy particle beams interact with the crystalline structure of optical fibers. In spatial irradiation, even electron beams with sufficiently high energy can induce displacement damage [39]. Although gamma rays can cause some displacement damage, their effect is minimal compared to particles like neutrons [40]. During elastic or inelastic collisions of high-energy particles with the lattice, if the lattice absorbs enough energy, the atoms within it will shift from their original positions and undergo rearrangement, leading to the formation of defects [41].

From a microscopic standpoint, displacement damage manifests through either elastic or inelastic collisions between incident particles or electrons and the network structure. When atoms absorb sufficient collision energy, they deviate from their original positions, forming interstitial atoms and vacancies, which, through cascade collisions, prompt rearrangements in the atomic structure. These interstitial and vacancy atoms induced by displacement damage may either recombine or further aggregate into defect clusters such as dislocation loops, voids, and stacking fault tetrahedra.

Displacement damage induces alterations in the glass network structure. Consider quartz glass, for instance, where the ground state energy level of silicon nuclei is 1.779 MeV and that of oxygen nuclei is 6.049 MeV. Only when the energy of incident particles reaches a sufficiently high level can the atomic nuclei attain higher excited states during collisions [40], resulting in atomic displacement. Inelastic collisions, prevalent in displacement damage, involve the transfer of energy from incident particles to atoms, causing atomic displacement and the formation of interstitial atoms and vacancies, known as non-paramagnetic defects [41]. This process can also trigger the displacement of oxygen atoms within the intrinsic structure of quartz glass, forming E’ centers [35].

Given that the energy required to induce displacement damage exceeds that of ionization damage, ionization damage stands as the primary mechanism behind radiation-induced damage in active optical fibers. Point defects stemming from this damage can lead to radiation-induced losses in fibers, thereby diminishing system gain and elevating background losses. At higher doses (>1 MGy) and dose rates, radiation-induced emission (RIE) and radiation-induced refractive index changes (RIRIC) may emerge, severely compromising the stability of optical systems.

#### 2.1.3. Color Center Defects

Color center defects arise from the ionization of outer atomic electrons and atomic displacement within optical fibers due to radiation exposure, leading to the generation of electron–hole pairs, atomic vacancies, and interstitial atoms [33,42]. Electron–hole pairs, carrying opposite charges, are easily captured by defects of opposite charge, thereby forming paramagnetic defects. Atomic vacancies and interstitial atoms cause the rearrangement of the atomic structure, resulting in the formation of new lattice structures. Additionally, defects formed by the rupture of chemical bonds under intense irradiation also fall into the category of paramagnetic defects [43].

#### 2.1.4. Point Defect Generation

In bulk glass and optical fibers, the properties of glass become more intricate due to the presence of intrinsic or extrinsic point defects induced during glass processing or specific treatment procedures. The defect structure relates to atoms with coordination deficiencies or excesses, substitution or interstitial impurities, or similar atoms (such as Si-Si or Si-O-O-Si) bonded within the silicon dioxide matrix. These defects correlate with absorption bands within the silica gap, resulting in reduced transparency of the glass or fiber. These defects may emerge during manufacturing processes, transform from existing centers under irradiation, or be directly generated by radiation from “perfect” positions.

Fused quartz boasts excellent optical properties. However, owing to its brittle nature, the mechanical processing of optical surfaces unavoidably leads to a significant number of surface defects, including pits, cracks, and scratches [44,45,46]. These surface defects substantially increase the absorption of optical components, thereby significantly diminishing their resistance to laser-induced damage under intense laser irradiation. It is widely believed that under the influence of mechanical force, point defects appear in the regions of surface defects [47]. Previous studies have demonstrated that point defects can serve as precursors to laser damage. Additionally, point defects are considered significant contributors to increased laser absorption and a decreased resistance of mechanically processed optical surfaces to laser-induced damage [48]. The density of point defects in surface defect regions closely correlates with the laser-induced damage threshold (LIDT) of mechanically processed optical surfaces, a notion widely accepted [49,50].

### 2.2. Macroscopic Effects of Radiation Damage

#### 2.2.1. Radiation-Induced Attenuation (RIA) mechanism

The macroscopic effects of radiation on the performance of optical fibers are specifically evident in three aspects: (1) radiation-induced attenuation (RIA), which affects the fibers’ transmission performance [51,52,53,54,55]; (2) radiation-induced emission (RIE), characterized by increased noise during fiber transmission, primarily observed when RIA is minimal [56,57]; and (3) influence on their optical and mechanical properties. The impact on the optical properties mainly manifests in changes to the fiber’s refractive index after irradiation. Irradiation can cause a reduction in the distance between silicon atoms within the fiber, leading to a decrease in silicon–oxygen bond angles, ion volume contraction, and an increase in the refractive index. The effect on the mechanical properties primarily arises from atomic structural rearrangement due to ionization and displacement damage caused by irradiation, resulting in alterations in the fiber density and volume [58,59,60,61,62].

Radiation-induced attenuation (RIA), affecting optical fibers exposed to radiation, is influenced by a multitude of factors: radiation dose (Gy) [63], dose rate (Gy/s) [64,65], radiation temperature [66,67], injected optical power [68], operating wavelength [69], composition of fiber core and cladding [51,70,71], fiber manufacturing processes [72,73,74], optical geometric parameters [75], and fiber optical conductivity [76].

During optical fiber irradiation at room temperature, it is common to observe an increase in RIA levels. However, after the irradiation ceases, most fibers experience a subsequent decrease in RIA levels, stabilizing at a nearly permanent level, dependent on the irradiation temperature. This behavior can be attributed to the generation of competing defects during irradiation and subsequent bleaching mechanisms post-irradiation. The primary parameters governing RIA effects in such fibers under irradiation conditions are the composition of the fiber core and cladding [77,78,79,80,81,82]. Consequently, current optimization strategies for RIA effects in optical fibers typically revolve around two approaches: one involves designing the fiber profile to alter its refractive index, while the other aims to ensure the fiber exhibits low attenuation levels. To achieve these goals, dopants are often introduced during the fiber manufacturing process, with common ones including germanium, fluorine, phosphorus, aluminum, nitrogen, erbium, and ytterbium [83].

In 2009, research employed polymethyl methacrylate (PMMA) fibers to monitor various radiation doses [84]. These fibers exhibited a linear response to radiation-induced attenuation across different wavelengths, consistent with the Beer–Lambert law. Due to PMMA plastic fibers’ inherent properties, they effectively monitored light between 500 nanometers and 700 nanometers. Sensitivity to radiation dose varied with wavelength, decreasing as the wavelength increased. Additionally, the dose range was found to be wavelength-dependent; while the fibers completely attenuated at lower wavelengths, higher wavelengths exhibited lower RIA sensitivity but still yielded measurable signals. Demonstrating excellent sensitivity, up to 0.6 dBm^−1^/kGy, the PMMA fibers could monitor dose ranges from 30 Gy to 45 kGy, surpassing the sensing capabilities of current sensors. By carefully selecting monitoring wavelengths, precise sensitivity and dose ranges can be achieved, allowing PMMA fibers to serve various applications from low-dose measurements in space and medical fields to high-dose measurements in the sterilization and nuclear industries.

Moreover, research explored gamma radiation responses based on fiber Bragg gratings (FBGs) [85]. Exposure to gamma radiation resulted in a shift in the Bragg wavelength (BW), correlating with the received dose. However, the sensitivity of the silica fiber Bragg gratings decreased with the increasing received dose, leading to reduced sensitivity. Additionally, their sensitivity was highly dependent on temperature.

#### 2.2.2. Densification Mechanism

Given the extensive applications of amorphous silica (α-SiO_2_) in fields like optical fibers and optical sensors, it has garnered significant attention from researchers [86]. The phenomenon of densification in α-SiO_2_, crucial in irradiation environments, has been extensively investigated. Research suggests that ionization or displacement damage induced by irradiation can trigger internal structural rearrangement or the formation of numerous defects within α-SiO_2_ materials. This process can result in the dissociation of intrinsic silicon–oxygen–silicon bonds, leading to an increase in the density of α-SiO_2_ materials. As the α-SiO_2_ density rises, so does its refractive index, thereby impacting its optical properties [87,88,89]. Consequently, this phenomenon has become a compelling subject for researchers in related fields. Buscarino et al. conducted further studies on the densification phenomenon of silica glass under irradiation environments [90]. They employed electron paramagnetic resonance (EPR) techniques to measure the 29Si hyperfine structure of E’γ centers. Their experimental findings suggested that structural alterations in the α-SiO_2_ under irradiation resulted from the nucleation of confined, highly defective, and densified regions dispersed statistically throughout the material’s volume.

## 3. Research Status of Radiation-Resistant Fiber Design

### 3.1. Radiation-Resistant Fluorine-Doped Fibers

In earlier studies on radiation-resistant optical fibers, undoped pure silica fibers were regarded as having the best radiation resistance until fluorine-doped fibers were developed [70,91,92]. Some researchers observed that fluorine-doped fibers showed remarkable radiation resistance. They proposed that incorporating fluorine atoms into the fiber’s cladding formed stronger bonds with silicon, thus reducing the fiber’s susceptibility to radiation [93,94,95]. As a result, fluorine-doped fibers have broad prospects for application in the sensor and communication fiber fields. Additionally, it is noteworthy that reports indicate that radiation detectors made from fully perfluorinated polymer optical fibers (PF-POFs) also exhibit outstanding performance [96,97].

Gusarov et al. successively investigated the transmission characteristics of cyclic transparent amorphous fluoropolymer (CYTOP) fibers exposed to gamma radiation doses of 1, 5, 20, and 50 kGy over an extended period (5 months after irradiation) [98]. They measured the changes in radiation-induced attenuation (RIA) for POF samples subjected to doses of 5 kGy and 17.5 kGy from 1–2 h to 80 h post-irradiation and compared them with long-term results. Their findings revealed that the fiber transmission partially recovered with the increasing irradiation time in the short-wavelength region (below 1000 nm), a phenomenon commonly observed in various types of fibers. Conversely, the RIA in the wavelength range above 1300 nanometers continued to rise post-irradiation and reached saturation, becoming permanent. The RIA value showed a close correlation with the time elapsed after irradiation. Furthermore, in dose determination applications, it is essential to consider the wavelength range; RIA stability requires time, but RIA values are permanent [99].

**Figure 1:**
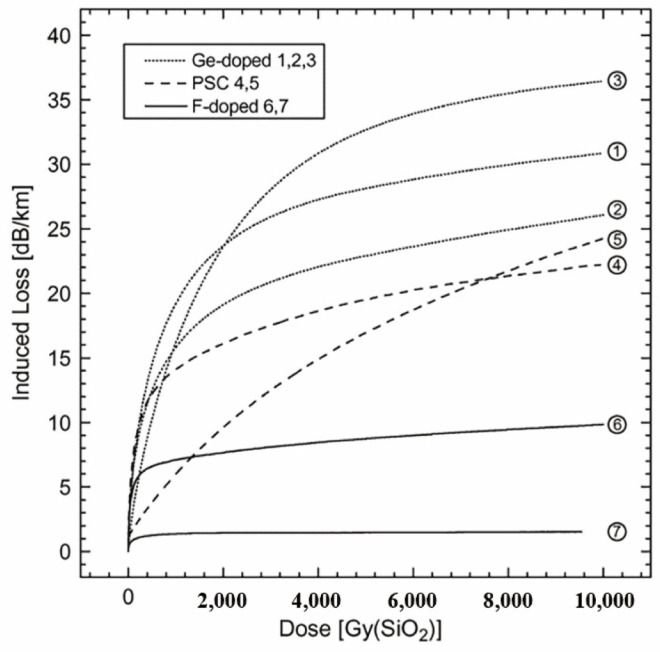
Comparative experiment by Wijnands et al. [100].

In 2008, Wijnands et al. published findings on the radiation resistance of fluorine-doped fibers [100]. Conducting irradiation experiments, they compared fluorine-doped fibers from Fujikura, Japan, with standard communication germanium-doped fibers and PSC fibers, as shown in Figure 1. The results revealed that fluorine-doped fibers displayed outstanding radiation resistance in both gamma radiation and high-energy-physics radiation environments.

**Figure 2:**
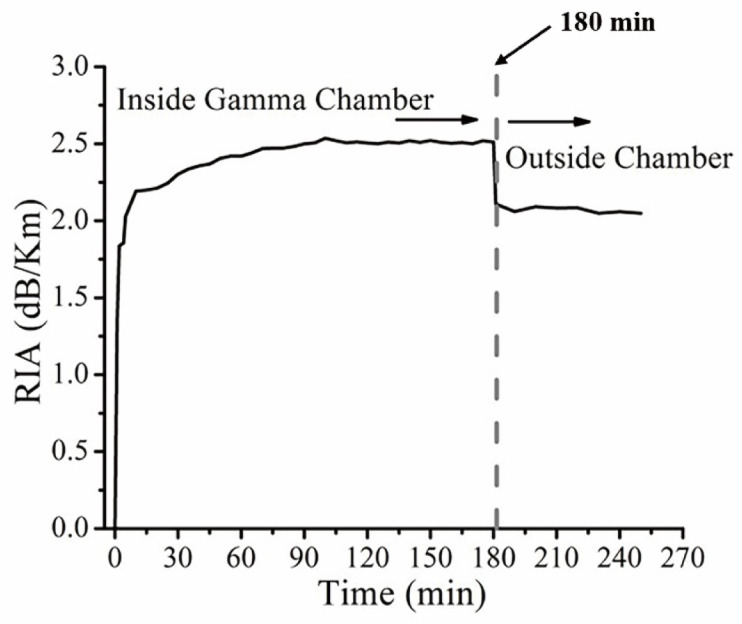
Radiation-induced attenuation (RIA) levels of silicon-core fluorine-doped cladding fibers used by Pal et al. before and after gamma irradiation [101].

In 2016, Pal et al. presented a radiation-resistant fiber Bragg grating sensor utilizing fluorine-doped fibers [101]. Through experiments, they discovered that silicon-core fibers with fluorine-doped cladding, produced using an improved Modified Chemical Vapor Deposition (MCVD) method, exhibited exceptional transmission and radiation-resistant properties in the near-infrared region. After 180 min of gamma radiation exposure, the radiation-induced attenuation (RIA) level of the silicon-core fluorine-doped cladding fibers used could rapidly recover to its pre-irradiation RIA level, as illustrated in Figure 2.

**Figure 3:**
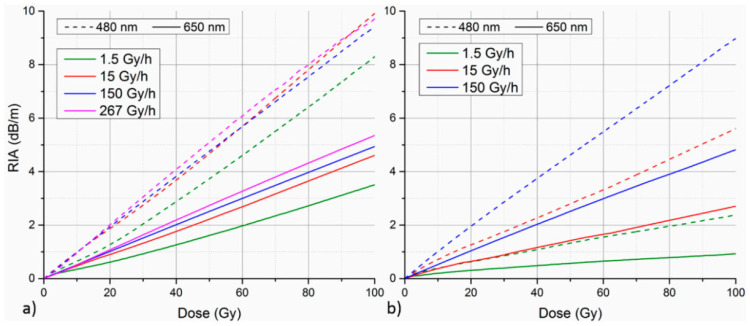
The variation curves of RIA with increasing irradiation dose for two types of PF-POFs compared in the experiment by Stajanca et al. [102]. (**a**) Copolymer-catalyst-type PF-POFs; (**b**) non-copolymer-catalyst-type PF-POFs.

In 2017, Stajanca et al. reported on the radiation-induced attenuation (RIA) levels of PF-POFs (perfluorinated polymer optical fibers) used for radiation monitoring [102]. They compared and evaluated the RIA levels of two commercial PF-POFs under gamma irradiation in the visible light spectrum and studied the influence of the irradiation dose rate and temperature on their RIA. At lower irradiation doses, both types of PF-POFs exhibited lower RIA levels, as shown in Figure 3. However, the copolymer-catalyst-type PF-POFs showed a lower dependence on the irradiation dose rate, with a greater increase in the RIA level as the irradiation dose increased. Therefore, copolymer-catalyst-type PF-POFs are more suitable for radiation monitoring applications, providing a new direction for developing novel radiation sensors.

While it is widely acknowledged that pure silicon-core fluorine-doped cladding silica fibers have the best radiation resistance, determining the optimal fluorine content and understanding the mechanism of fluorine’s action remain unclear. Consequently, fluorine-doped fibers continue to be a research direction that requires further development.

In 2017, Blanc et al. conducted a study on the attenuation behavior of two types of radiation-resistant fluoride-doped single-mode optical fibers under room-temperature (297 K) and low-temperature (16 K) conditions at wavelengths of 1312 nm and 1570 nm [103]. Apart from a notable increase in optical attenuation at 16 K, it was observed that the fibers in the frozen state ceased to undergo thermal bleaching and defect recombination. However, prolonged recovery, including heating the fibers from low temperature to room temperature, facilitated the annealing of numerous defects, resulting in an almost complete restoration of the fibers to their initial performance. As the fibers were in a frozen state at 16 K, the primary mechanism for defect recovery was inhibited, leading to a significant rise in radiation-induced attenuation. At room temperature, the tested F-doped fibers exhibited only a few dB/km of radiation-induced attenuation, whereas at low temperature, the radiation-induced attenuation at 1312 nm surpassed 600 dB/km, with a total induced dose of 10 kGy. Nonetheless, despite employing a two-step annealing process, the fibers recovered over 90% of their performance, although some residual attenuation persisted. These findings underscore the importance of considering optical bleaching effects, typically deemed negligible at room temperature, even at low temperatures. Additionally, it is well established that optical fibers demonstrate a “memory” effect, wherein radiation-induced attenuation rapidly returns to values close to pre-annealing levels upon radiation recovery. Further investigation into this phenomenon, particularly under low-temperature conditions, is warranted to elucidate the role of annealing.

### 3.2. Radiation-Resistant Erbium-Doped Fibers

Erbium-doped fibers (EDFs) find widespread applications in the aerospace industry as laser sources and optical amplifiers [104]. In the space environment, where such equipment is frequently exposed to high-energy radiation, ensuring radiation resistance becomes a critical consideration during their design phase [105,106]. Studies have revealed that compared to traditional pure silica-core fibers and germanosilicate fibers, EDFs exhibit superior resistance to radiation [107].

In 1992, G. M. Williams et al. published research on radiation effects in EDFs [108]. Their experiments demonstrated that doping Er^3+^ ions into the fiber core, along with aluminum and germanium, resulted in enhanced radiation-induced darkening compared to conventional germanium-doped fibers. The extent of the darkening may be correlated with the stoichiometry of Er^3+^/Al_2_O_3_.

In 2001, Todd S. Rose et al. reported their investigation into the radiation sensitivity of erbium-doped fibers (EDFs) used in low-noise optical amplifiers for space applications [109]. Due to the longer fiber paths of EDFs used in space and the sensitivity enhancement resulting from Er^3+^ doping, even low doses of radiation can lead to significant signal attenuation. Moreover, in space environments, minor fluctuations in the front-end inversion of low-noise amplifiers (LNAs) can cause substantial variations in the noise figure (NF), thereby severely impacting fiber performance. Hence, understanding the mechanism underlying the degradation of fiber performance caused by radiation becomes crucial.

The team simulated high-energy radiation in space using gamma rays and measured both the passive transmission of EDFs and the gain and noise figure in active amplifiers. Their experiments revealed that the performance degradation of EDFs is primarily attributed to the absorption of 980 nm pump light induced by radiation. This issue can be mitigated by using sufficiently high pump power levels in the amplifier to achieve inversion saturation. Additionally, operating the amplifier in a pumped state can induce continuous optical annealing in the fiber, thereby alleviating performance degradation.

In 2007, S. Girard et al. reported a study on the impact of proton- and gamma-induced effects on erbium-doped fibers (EDFs) [110]. By measuring the radiation-induced attenuation (RIA) from the visible range to the infrared range, the group characterized the proton- and gamma-induced effects on three types of EDFs. The doping of Er^3+^ in the fibers was enhanced by controlling the doping level of Al_2_O_3_ in Ge-P silica fibers (Table 1). The changes in the RIA compared to the best linear fit of the RIA at 980 nm versus dose D (in Gy) were measured with two different energy levels (50 MeV and 105 MeV) of protons or gamma rays (Table 2). It was observed that the fiber performance degradation decreased consistently from the visible spectrum to the infrared spectrum. Using these measurements, the RIA curves were spectrally decomposed, revealing that the RIA phenomenon in EDFs is mainly correlated with the doping level of aluminum, while the presence of Er^3+^ ions appears unaffected by proton radiation. This may be attributed to the formation of a protective solvated shell around Er^3+^ by Al_2_O_3_, offering shielding against proton radiation.

In 2009, A. Gusarov et al. reported on the radiation sensitivity of amplifiers utilizing highly doped Er^3+^ fibers [111]. The group employed nano-deposition techniques to fabricate several erbium-doped fiber amplifiers (EDFAs) with similar gain characteristics. They utilized a ^60^Co γ-radiation source and measured the small-signal gain and noise figure during post-radiation annealing processes. Significant performance degradation was observed in these fibers. However, when the fiber gain remained sufficiently high, exceeding 10 dB, the reduction in the noise figure was negligible. The EDFA with the lowest Er^3+^ concentration exhibited the most pronounced performance degradation, primarily due to increased fiber background absorption induced by radiation. Increasing the Er^3+^ concentration allowed for shortening the fiber length, thereby reducing radiation-induced background absorption and enhancing the fiber’s radiation resistance. However, excessively high Er^3+^ concentrations required additional dopants to prevent Er^3+^ ion clustering, which, in turn, exacerbated the degradation of the fiber performance.

In the same year, Li et al. reported a study on the radiation effects of EDFAs with different densities [112]. Under the irradiation of a ^60^Co γ-radiation source at the same rate and total radiation dose, amplifiers using EDFs with two different densities were able to achieve the same output power. Experimentally, it was found that in the irradiation environment, the length factor of the EDF had a greater impact on the radiation sensitivity of the fiber compared to the density factor. In other words, under the same output power conditions, the performance of short EDFs with a high Er^3+^ concentration was superior to that of long EDFs with a low density. This experimental result further confirms that the Er^3+^ concentration is the primary factor affecting the performance of EDFs in radiation environments.

In 2012, Thomas et al. reported on a highly radiation-resistant erbium-doped pure silica fiber containing erbium-doped nanoparticles [113]. The group initially compared the radiation resistance of five different chemically composed erbium-doped fiber amplifiers (EDFAs) under irradiation from a ^60^Co γ-radiation source. They measured the optical gain and noise figure in saturated amplifiers as well as the radiation-induced attenuation (RIA) at pump and signal wavelengths in the small-signal state. The experimental findings revealed that EDFAs doped with aluminum, whether conventional or nanoparticle-based, exhibited a higher gain degradation and RIA. Conversely, nanoparticle-based EDFAs without aluminum doping showed a lower gain degradation and RIA.

Previous studies on EDFs have primarily focused on high-dose irradiation conditions, generally suggesting that EDFs’ radiation-induced attenuation (RIA) increases with higher irradiation doses [114,115]. However, some reports indicate an anomalous phenomenon known as Enhanced Low-Dose-Rate Sensitivity (ELDRS) in EDFs under low-dose irradiation conditions [116]. In 2012, Gilard et al. presented a theoretical framework model to explain ELDRS in EDFs [117]. The group proposed that the competition between recombination centers and charge trapping in silica might be the primary factor contributing to ELDRS. Additionally, the observed dose and dose rate ranges of ELDRS are significantly influenced by the recombination kinetics of the fiber carrier. It is noteworthy that under specific irradiation dose conditions, the presence of deep fiber defects and dispersed transmission, limiting de-trending occurrence, often reduces the dose rate threshold for ELDRS. Hence, in low-dose-rate environments (such as on the ground), some conventional fibers with dose rate sensitivity, like germanium-doped fibers and pure silica fibers, do not exhibit the ELDRS phenomenon due to their low recombination center density and high dose rate threshold. In contrast, EDFs, with their higher recombination center density and lower dose rate threshold, are more susceptible to ELDRS.

In 2014, S. Girard et al. introduced a novel radiation-resistant erbium-doped fiber amplifier (EDFA) [118]. Unlike conventional EDFAs utilizing standard EDFs, this new type of EDFA employs Hole-Assisted Carbon-Coated (HACC) EDFs. The unique HACC structure enables the fiber core to achieve optimal gas loading (with either H2 or D2) while reducing its radiation sensitivity without compromising the performance of the EDFA.

In 2021, Yan et al. systematically investigated the spectral properties and radiation resistance behavior of Er/Al/Ge co-doped silica glass and fibers under the influence of germanium dioxide [119]. The team further elucidated the principle of color center formation in the glass and fibers under gamma-ray irradiation and speculated on the mechanism behind the radiation hardening. The experimental results confirmed that co-doping with Er/Al/Ge did not affect the spectral properties of the Er^3+^ and significantly enhanced the radiation resistance of the glass and fiber samples. Moreover, the degree of performance enhancement was directly proportional to the content of germanium dioxide in the glass and fibers.

In summary, research on EDFs has matured, with a well-developed understanding of related mechanisms being obtained, indicating substantial potential for further development.

### 3.3. Radiation-Resistant Ytterbium-and-Erbium Co-Doped Fibers

Research on ytterbium-doped fiber (YDF) has a history spanning several decades. Thanks to its wide gain bandwidth, long upper-state fluorescence lifetime, low quantum defects, and high quenching concentration, optical amplifiers made with YDF, known as erbium-doped fiber amplifiers (EDFAs), offer higher output power. Consequently, mature EDFA technology has become one of the mainstream amplifiers used in ground-based communications [117,120,121]. With the increasing demand for space communication, high-performance EDFAs have attracted attention from researchers [122]. Previously, EDFAs were mainly used in ground-based communications, which differ from space environments with more severe radiation conditions. Therefore, developing radiation-resistant YDF has become a focus of researchers, with significant achievements, particularly in the field of erbium–ytterbium co-doped fibers (EYDFs) [113,123].

In 2009, Li et al. reported experimental studies on the radiation effects of erbium–ytterbium co-doped fiber amplifiers (EYDFAs) for space optical communication in low-dose-radiation environments [124]. Using ^60^Co as the radiation source, EYDFAs were irradiated at a total dose of 50 krad with a dose rate of 40 krad/s. Measurements were conducted on the wavelength peak and full width at half maximum (FWHM) of the fiber signal. The results indicated no significant changes in the wavelength peak and FWHM at five experimental breakpoints, demonstrating the stability of EYDFAs for communication in radiation environments.

**Figure 4:**
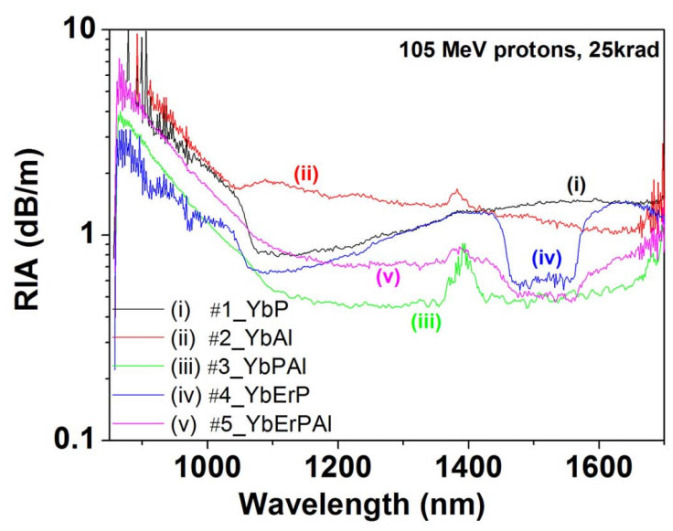
RIA of the five types of fibers measured by S. Girard et al. under irradiation conditions [125].

In the same year, S. Girard et al. reported their study on the effects of YDF and EYDF under irradiation [125]. Through exposure to 105 MeV proton and gamma-ray irradiation tests on five different fiber groups (YbP, YbAl, YbPAl, YbErAl, YbErPAl), the RIA was measured, as shown in Figure 4. The experimental results indicate that, compared to YDF, except for ytterbium–phosphorus–aluminum co-doped fibers, EYDF exhibits better spectral performance under irradiation, demonstrating that EYDF is an excellent candidate material for radiation-resistant EDFAs.

In 2018, Ladaci et al. reported on the changes in the spectral properties of Er^3+^ and Yb^3+^ ions in phosphate–silicate-based fiber matrices under X-ray, γ-ray, electron, and proton irradiation [28]. The experimental results indicated that both Er^3+^ and Yb^3+^ ions exhibited a significant decrease in their infrared emission lifetimes with increasing irradiation dose. Specifically, the emission lifetime of the Er^3+^ ion’s ^4^I_13/2_ level decreased from approximately 9 ms before irradiation to around 6.3 ms, while that of the Yb^3+^ ions’ ^2^F_5/2_ level decreased from about 1.8 ms to approximately 0.65 ms before irradiation. Additionally, Ladaci et al. also tested the RIA changes after doping cerium ions into EYDF. The experimental results showed that the inclusion of cerium ions not only enhanced the RIA tolerance of the EYDF but also reduced the impact of radiation on the Er^3+^ ions’ ^4^I_13/2_ level, providing a new avenue for developing radiation-resistant EYDF.

In summary, compared to traditional YDF, EYDF exhibits better radiation resistance and is more suitable for use in the harsh radiation environment of space. However, there are relatively few reports on the mechanism of EYDF, and further research on the relevant radiation-resistant mechanisms of EYDF is still needed for its commercial application.

### 3.4. Radiation-Resistant Nitrogen-Doped Fibers

There is limited research on radiation-resistant nitrogen-doped fibers, but existing reports suggest that core-doped nitrogen-doped silica fibers show promising performance in radiation resistance. Some studies even propose them as highly potential materials for optical-induced fiber Bragg and long-period grating coupling.

**Figure 5:**
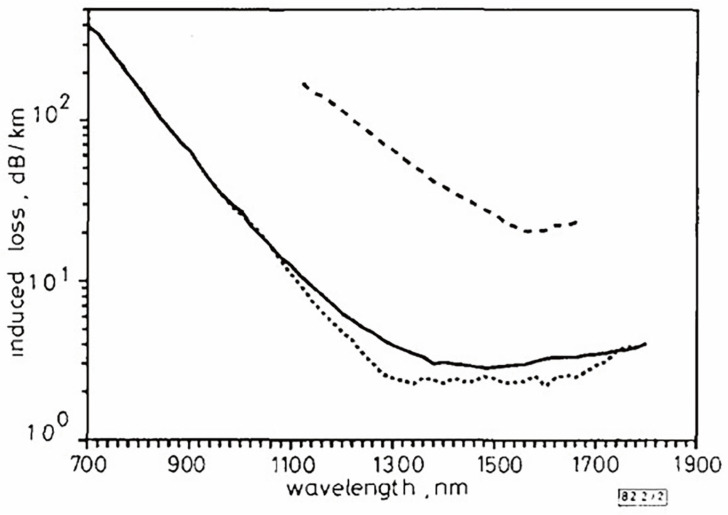
The experimental results of radiation-induced losses in radiation-resistant core-doped nitrogen-doped silica fibers tested by Diano et al. Solid line represents nitrogen-doped silica core fiber under investigation; Dash line represents MCVD single-mode pure silica core fluorine-doped silica cladding fiber; Densely dash line represents MCVD single-mode germanium-doped silica core fluorine-doped silica cladding fiber [126].

In 1995, Dianov et al. reported on a radiation-resistant core-doped nitrogen-doped silica fiber [126]. Under gamma-ray irradiation of 10 kGy for 1–2 h, the experimental results showed that the radiation-induced losses of the core-doped nitrogen-doped silica fiber in the range of 1300–1600 nm were very close to the optimal data from previous radiation experiments on pure silica fibers, as shown in Figure 5. This study suggests that core-doped nitrogen-doped silica fibers hold promise as radiation-resistant fibers.

Optical fiber Bragg gratings (FBGs) and long-period grating (LPG) sensors within radiation-resistant core-doped nitrogen-doped silica fibers are promising candidates for sensor applications in nuclear and other radiation environments [127,128]. These materials demand high radiation resistance from the optical fibers employed [129,130]. In 1998, Vasiliev et al. reported on the performance of FBGs and LPGs made from core-doped nitrogen-doped silica fibers under gamma-ray irradiation [131]. The experimental results demonstrated the excellent radiation resistance of both the FBGs and LPGs fabricated using core-doped nitrogen-doped silica fibers under a dose of 1.46 MGy gamma-ray irradiation.

In 2004, S. Girard et al. reported on the irradiation effects of core-doped nitrogen-doped silica fibers under gamma-ray and pulsed X-ray irradiation [132]. Throughout most of the irradiation experiments, the core-doped nitrogen-doped silica fibers exhibited minimal radiation-induced losses at both 1.55 μm and 1.31 μm wavelengths. Additionally, the core-doped nitrogen-doped silica fibers demonstrated outstanding radiation resistance at 1.55 μm, comparable to the best experimental data for pure silica fibers.

In summary, nitrogen-doped fibers have been shown to exhibit better radiation resistance compared to pure silica fibers. However, there is still limited research on their underlying mechanisms, and further studies are needed for nitrogen-doped fibers to realize their full potential in practical applications.

### 3.5. Radiation-Resistant Germanium-Doped Fibers

Ge-doped fibers are not the primary focus of research in radiation-resistant fiber optics. Some researchers argue that germanium ions are one of the primary causes of radiation-induced defect structures in conventional communication-grade silica fibers [133]. Ge replaces silicon in the silica tetrahedron, causing distortion in the regular tetrahedral structure within the fiber and generating internal stress, ultimately leading to defect formation. Additionally, these impurity atoms have a strong electron affinity, easily capturing the charges generated after irradiation to form color centers, which absorb specific wavelengths of light signals and increase losses [134]. S. Girard et al. studied the radiation resistance of pure silica-core and Ge-doped silica-core fibers in steady-state gamma and pulsed X-ray irradiation environments [135]. A comparison revealed that the radiation resistance of pure silica-core fibers is significantly better than that of Ge-doped silica-core fibers, and the performance of Ge-doped silica-core fibers is mainly determined by the material of their cladding. T. Shikama et al. investigated the effects of F and Cl on the radiation resistance of Ge-doped silica fibers [136,137]. The results indicate that both can effectively suppress light absorption related to Ge at 240 nm and the corresponding intensity of the ESR spectrum, suggesting that F and Cl are beneficial for improving the radiation resistance of Ge-doped silica fibers. However, some researchers believe that Ge-doped fibers can be applied in certain specialized fields.

In 2013, Benabdesselam et al. reported on the performance of germanium-doped fibers as thermoluminescent dosimeters for luminescence radiation dosimetry [138]. Through experiments, the dose–response characteristics of three germanium-doped fiber dosimeters under X-ray irradiation were compared, and the capabilities of these dosimeters were evaluated for monitoring different doses and dose rates of gamma rays as well as different particles (0.8 and 14 MeV neutrons and 63 MeV protons). The experimental results demonstrated that the dosimeters made from germanium-doped fibers exhibited good neutron and proton detection efficiency and high sensitivity to gamma radiation. Moreover, the position of the dosimetric peaks storing information about the absorbed dose on the thermally stimulated luminescence (TSL) growth curves of the three dosimeters was nearly perfect, being both high enough to measure absorbed dose at high temperatures and low enough to fully determine the absorbed dose. Therefore, germanium-doped fibers have great potential for applications in TSL dosimetry, meeting all the major standards for clinical dosimetry requirements in the medical field and providing superior performance compared to two commercially available TSL dosimeters. Additionally, germanium-doped fibers exhibit a good linear response to photons and show no dose rate dependence, suggesting potential applications in the field of physics. It is worth mentioning that germanium-doped fibers can quantify the single-energy radiation flux of neutrons and protons and, if their energy distributions are known, can also be used for neutron spectrum measurements.

In 2021, Eronyan et al. reported on a 20% germanium-doped elliptical-core fiber embedded in a Germanosilicate Optical Fiber with an Elliptical Core (GOFEC) [139]. Through experiments conducted at temperatures of 25 °C and −60 °C with a wavelength of 1550 nm and a dose rate of 1 Gy/s of gamma radiation, the radiation resistance of the GOFEC was investigated. The experimental results indicated that the GOFEC exhibited excellent radiation resistance, particularly outperforming most existing fibers at −60 °C.

In summary, the current research developments indicate that germanium-doped fibers are only applicable in certain specialized fields, making them not the mainstream direction for radiation-resistant fibers.

### 3.6. Radiation-Resistant Cerium-Doped Fibers

Cerium is commonly used to manufacture radiation-resistant glasses. In these glasses, trivalent and tetravalent cerium ions undergo oxidation–reduction reactions by capturing electrons and holes generated during irradiation. Trivalent cerium ions capture holes, inhibiting the formation of hole-capturing color centers, while tetravalent cerium ions suppress the formation of electron-capturing color centers, effectively enhancing the glass’s radiation resistance [140,141,142]. This has prompted some researchers to explore cerium doping in optical fibers to enhance their radiation resistance.

In 2016, Francesca et al. conducted a study on the effects of cerium doping on the radiation resistance of germanosilicate and phosphosilicate optical fibers [57]. The experimental findings revealed that the cerium doping did not improve the radiation resistance of the germanosilicate optical fibers in the ultraviolet-visible range but did enhance the radiation resistance of the phosphosilicate optical fibers within the same range. Electron paramagnetic resonance (EPR) spectroscopy confirmed that the cerium played a pivotal role in determining the type and density of the radiation-induced defects. Regardless of whether it was the germanosilicate or phosphosilicate optical fibers, the cerium ions acted as electron donors under irradiation, significantly reducing the number of radiation-induced hole centers. Therefore, cerium-doped optical fibers with enhanced radiation resistance show considerable potential for further research and development.

### 3.7. Radiation-Resistant Aluminum-Doped Fibers

Aluminum plays a crucial role in optimizing amplifier performance and is commonly used in the production of rare-earth-doped optical fibers [143]. However, compared to pure silica fibers, aluminum-doped optical fibers exhibit increased sensitivity to radiation-induced attenuation (RIA) [144,145,146]. Therefore, in order to enable the application of aluminum-doped optical fibers in harsh radiation environments, some researchers have conducted studies on the radiation effects of these fibers.

In 2018, Alessi et al. reported on the influence of radiation on aluminum silicate fibers [147]. They conducted online X-ray RIA experiments on aluminum silicate fibers with different aluminum concentrations and observed absorption bands associated with aluminum defects in the UV–visible spectrum. Interestingly, these bands were found to be independent of the radiation dose rate. Furthermore, factors such as the aluminum content in the preform materials, core size, manufacturing processes, and drawing parameters did not significantly affect the RIA levels and kinetics of the fibers. Notably, Alessi et al. delved into the mechanism of radiation effects on aluminum silicate fibers and confirmed the presence of Aluminum–Oxygen Hole Centers (Al–OHC) through their 2.3 eV absorption band and electron paramagnetic resonance (EPR) characteristics. They demonstrated that the growth kinetics of the Al–OHC concentration was linearly correlated with the radiation dose, but a significant saturation of the EPR data occurred at higher doses, indicating a precursor reaction in the formation process of the Al–OHC.

In 2021, Alessi et al. further investigated the radiation effects on aluminum silicate fibers [148]. They conducted online X-ray RIA experiments on these fibers in the near-infrared (NIR) range to assess their dosimetric potential. The growth rate of the RIA remained constant as the radiation dose rate increased from 0.073 to 6.25 Gy(SiO_2_)S-1. However, when the dose reached 2 kGy(SiO_2_), the RIA began to linearly increase. Additionally, at a radiation temperature of 50 °C, small but significant changes in the RIA were observed during continuous irradiation. This phenomenon is particularly important for the application of aluminum silicate fibers as dosimeters, as it can affect the accuracy of measurement results. Furthermore, through spectral analysis, Alessi et al. found no significant dependence between the fibers’ spectral shape and radiation parameters. Therefore, the data obtained at 1310 nm and 1550 nm not only provide dynamic information on the RIA for telecommunications and sensor fibers but also serve as a reference for commonly used NIR ranges in related fiber technologies.

### 3.8. Radiation-Resistant Phosphorus-Doped Fibers

Research into using the radiation-induced attenuation (RIA) effect of optical fibers for radiation dosimeters has been ongoing for several decades [149,150]. Phosphorus doping in optical fibers has been shown to significantly enhance fiber RIA sensitivity to radiation, enabling the fabrication of high-performance radiation dosimeters [151,152]. Both pure phosphorus-doped fibers and germanium/phosphorus co-doped fibers have been demonstrated to manufacture dosimeters suitable for use in high-radiation-dose environments [153].

In 2013, Gusarov et al. compared the γ-radiation-induced attenuation effects among phosphorus-doped, aluminum-doped, and germanium-doped optical fibers at irradiation temperatures ranging from 30 °C to 80 °C using ^60^Co as the γ-radiation source [153]. The experiments revealed that, compared to the germanium-doped fibers, the phosphorus-doped and aluminum-doped fibers exhibited superior radiation sensitivity, meeting dosimetric requirements. Additionally, as the irradiation temperature increased from 30 °C to 80 °C, the fibers doped with different materials showed varying responses in RIA levels with temperature. The absorption level of the aluminum-doped fibers decreased by 25%, while that of the phosphorus-doped fibers increased by 10%, indicating the excellent potential application of phosphorus-doped fibers in radiation dosimeters.

In 2018, Francesca et al. investigated the effect of irradiation on phosphorus-doped fibers at different temperatures [154]. Within the temperature range from 25 °C to 100 °C, there was a slight increase in damage induced by irradiation in the super-visible range. However, as the temperature exceeded 150 °C up to 280 °C (the highest temperature achievable under the experimental conditions of Francesca et al.), the RIA induced by irradiation in the same spectral range decreased with increasing temperature. Notably, Francesca et al. also studied the combined effect of ionizing radiation and irradiation temperature on phosphorus-doped fibers at doses of 3 MGy using online RIA measurements and post-mortem EPR measurements at irradiation temperatures ranging from 25 °C to 280 °C. Both the RIA and EPR data indicated that increasing the irradiation temperature led to an increase in point defects and associated absorption bands, providing important theoretical references for the future development of phosphorus-doped fibers for applications in radiation environments.

In 2019, Francesca et al. reported further research on single-mode phosphosilicate optical fibers [155]. Through experiments involving irradiation with ^60^Co γ-rays, X-rays, and protons, various characteristics of single-mode phosphosilicate optical fibers in irradiation environments were studied: (1) the fibers’ RIA strictly monotonically depends on the radiation dose (linearity up to 500 Gy); (2) the fibers’ RIA does not recover after the irradiation ends; (3) the fibers’ RIA does not depend on the radiation dose rate; (4) the fibers’ irradiation sensitivity is independent of whether the fibers have been previously irradiated; (5) the fibers exhibit stable resistance to photobleaching; and (6) within the irradiation temperature range from 20 °C to 45 °C, the fibers’ performance shows no temperature dependency. Considering these characteristics, Francesca et al. concluded that the single-mode phosphosilicate optical fibers used in the experiments are highly suitable for applications in Total Ionizing Dose (TID) sensors and Distributed Optical Fiber Radiation Sensors (DORFS). Moreover, they are fully compatible with commercially available single-mode OTDRs. Additionally, these fibers will be selected for use in six dispersion suppressor regions of the Large Hadron Collider (LHC) at the European Organization for Nuclear Research (CERN) and in three circular accelerators of the LHC injector chain: the proton synchrotron booster, the proton synchrotron, and the super proton synchrotron’s DORFS.

In 2020, Vecchi et al. demonstrated the reusability of near-infrared RIA using phosphosilicate optical fibers for point or distributed dose measurements in irradiation environments [156]. By injecting a continuous visible laser into the fibers and utilizing the photobleaching (PB) effect to regenerate the fibers, the most effective PB was achieved with a laser injection at 405.5 nm. Under these conditions, using a 1 m long phosphosilicate optical fiber irradiated with 1170 Gy after 1 h of laser injection at 5 mW, an approximately 75% recovery rate was achieved. When using a 30 m long phosphosilicate optical fiber irradiated with 100 Gy after the injection of a 514 nm laser at 430 mW, a recovery rate as high as 97% was attained. These results demonstrate, for the first time, the possibility of regenerating phosphorus-doped optical fiber dosimeters in the infrared region. Importantly, during this regeneration process, the fibers’ irradiation sensitivity remained unchanged, indicating that under the same optical response conditions, phosphorus-doped fiber dosimeters can reset the sensor for successive applications. This technology can effectively prolong the sensor’s lifespan and reduce maintenance costs.

### 3.9. Influence of the Fiber Structure on Its Radiation Response

Commercial optical fibers are available in various categories: Commercial Off-The-Shelf (COTS) multimode (MM) or single-mode (SM) fibers. Despite their similar optical and structural properties before irradiation, the different categories of fibers can exhibit markedly different radiation sensitivities. Most previous studies have focused on fibers based on Total Internal Reflection (TIR) technology to ensure guided light propagation. These fibers typically feature a silica core surrounded by polymer or metal coatings. The doping methods employed for different signal transmission media vary to ensure that the resulting Radial Index Profile (RIP) effectively guides the light.

**Figure 6:**
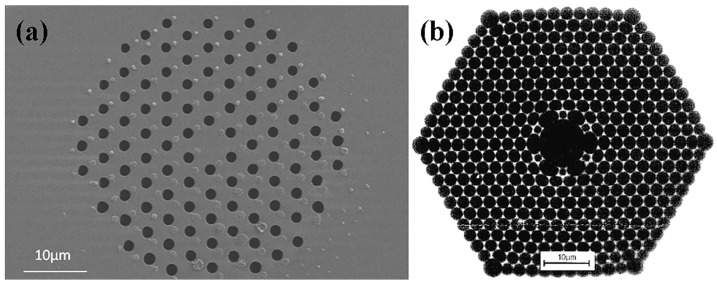
Schematic diagrams of (**a**) TIR microstructured fiber [157] and (**b**) Hollow-Core Fiber (HCF) [158].

Microstructured or photonic crystal fibers are increasingly utilized for sensor applications [157,158]. Two distinct types of fibers have been commercialized. The first type comprises TIR solid-core fibers with microstructured cladding, as illustrated in Figure 6a. The second type are Hollow-Core Fibers (HCFs), as illustrated in Figure 6b. HCFs consist of a core with air holes and a microstructured cladding. The light-guiding mechanism of these fibers differs significantly from TIR fibers, resulting in narrow transmission windows centered around wavelengths determined by the structural parameters.

Research findings regarding the radiation response of microstructured fibers are currently limited. For TIR solid-core fibers, their susceptibility to radiation appears to be comparable to that of all-silica fibers made from the same glass. In contrast, Hollow-Core Fibers (HCF) exhibit higher radiation resistance under steady-state gamma-ray irradiation, but their transient radiation response under pulsed X-rays is more complex. Apart from their unique optical properties, one advantage of these fibers is their ability to be manufactured using only one type of glass, reducing the complexity of the fiber’s radiation response. Additionally, the unique guiding properties of HCFs can be utilized for hardened waveguides, enabling adaptation to radiation environments and leading to compaction phenomena within the silica.

### 3.10. Radiation-Resistant Fibers Fabricated by a Pretreatment Method

Many studies have demonstrated that the radiation resistance of optical fibers can be significantly enhanced through the use of specialized pretreatment methods, including gas loading, pre-irradiation, and dehydration.

**Figure 7:**
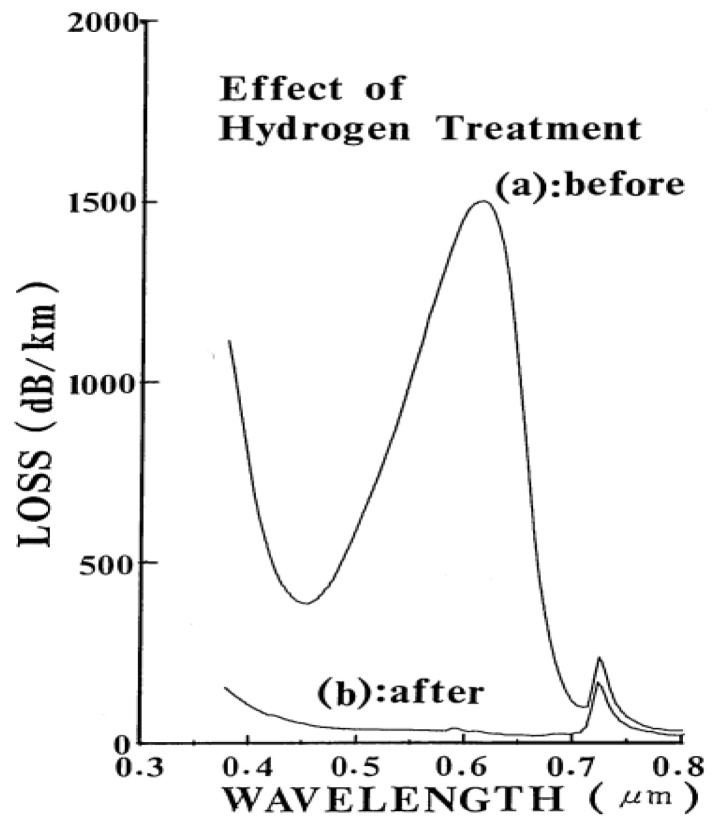
A comparison of radiation-induced losses before and after hydrogen gas pretreatment in the optical fibers used by Nagasawa et al. [159].

In 1985, Nagasawa et al. proposed a method to improve the radiation resistance of pure silica optical fibers through hydrogen loading pretreatment [159]. When subjected to gamma-ray irradiation, pure silica optical fibers with glass cladding exhibit notable absorption in the visible light range. This phenomenon can be particularly detrimental for image-guiding fibers, as it can substantially affect the fiber transmission. Such absorption may arise from non-bridging oxygen defect centers induced by irradiation or oxygen radicals resulting from chemical bond breakage. However, pretreating pure silica optical fibers with hydrogen gas can effectively mitigate this radiation-induced absorption phenomenon, as illustrated in Figure 7.

In 1996, Griscom reported a method to enhance the radiation resistance of optical fibers through pre-irradiation treatment [160]. Pre-irradiating pure silica optical fibers with gamma rays greater than 1 MGy can effectively reduce the radiation-induced attenuation in the visible light spectrum under gamma-ray irradiation. This attenuation in the visible light range is primarily caused by radiation-activated impurities (mainly chlorides) and precursor formation of non-bridging oxygen bonds in the fiber. When the concentration is less than 100 ppm, these precursor defect centers undergo chemical neutralization reactions with oxygen molecules under gamma-ray irradiation, thereby reducing the radiation-induced absorption. Griscom’s experimental results showed that the pre-irradiation treatment of pure silica optical fibers resulted in a hardening level of about 10 dB/km at 610 nm, with the level exponentially decreasing with the increasing pre-irradiation dose, indicating the potential for the further hardening of pure silica optical fibers.

**Figure 8:**
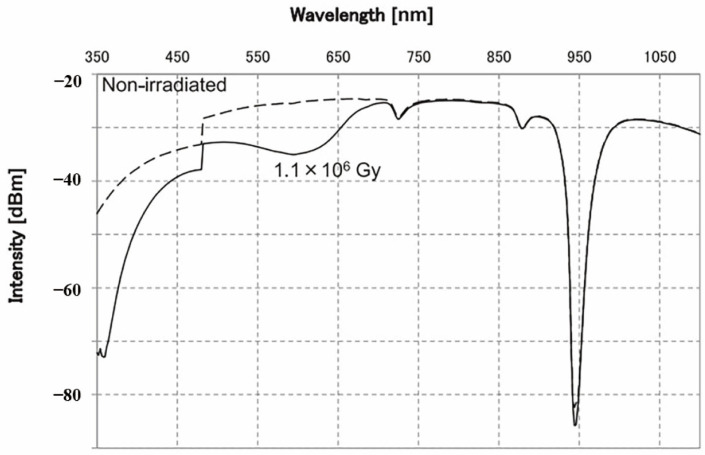
Wavelength spectrum of transmitted optical power using fibers in the experiment conducted by Ito et al. [161].

**Figure 9:**
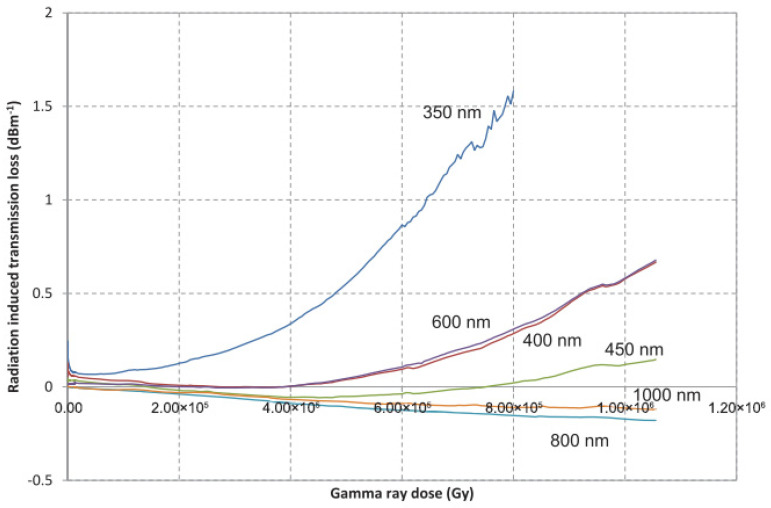
Radiation-induced transmission loss of fibers used in the experiment conducted by Ito et al. [161].

In 2014, Ito et al. presented a method to significantly enhance the radiation resistance of optical fibers by meticulously eliminating water during fiber manufacturing and elevating the hydroxyl concentration inside the fiber to 1000 ppm [161]. Utilizing ^60^Co as the gamma radiation source, the fibers employed in the experiment exhibited minimal absorption in the infrared region, as shown in Figure 8, coupled with a noticeable reduction in the radiation-induced transmission loss within the range from 600 nm to 800 nm, as shown in Figure 9. These findings suggest that fibers processed using this method demonstrate outstanding radiation resistance. Furthermore, Ito et al. speculated that the mechanism behind this approach might involve the increase in the hydroxyl concentration within the fiber, consequently reducing the initial concentration of precursors responsible for color center formation and thereby mitigating radiation-induced damage caused by color center defects.

In 2020, Shao et al. introduced a novel pre-treatment method for YDF consisting of three steps: deuterium loading, pre-irradiation, and thermal annealing, which effectively enhances the radiation resistance of YDF [162]. Through irradiation experiments, they compared the influence of the deuterium loading pre-treatment on the optical loss at 1200 nm and the laser refractive index of YDF under gamma-ray irradiation, demonstrating a significant improvement in the radiation resistance of YDF using this pre-treatment method. Vacuum experiments confirmed that YDF treated with this method maintains stable radiation resistance in a vacuum environment. Shao et al. also investigated the mechanism behind this pre-treatment method. CW-EPR spectra showed that the pre-treated YDF effectively suppressed the formation of color centers, and Raman and Fourier transform infrared (FTIR) spectra confirmed that the reduction in color center formation may be due to the inhibition of color center precursors by deuterium radicals.

In 2022, Jiao et al. reported a similar pre-treatment method using deuterium loading, pre-irradiation, and thermal annealing, effectively enhancing the radiation resistance of EDF [163]. Further investigation into the mechanism by Jiao et al. revealed that the pre-treatment method resulted in the formation of new chemical bonds (such as OD-) within the fiber, as evidenced by FTIR and absorption spectra. RIA and EPR spectra indicated a significant decrease in the concentration of radiation-induced color centers after the pre-treatment, attributed to the presence of OD- functional groups and D- radicals. However, there was also a decrease in the hydroxyl concentration, which may not be conducive to reducing the initial concentration of precursors that lead to color center formation.

In summary, the current pre-treatment methods for radiation-resistant fibers have matured, with deuterium loading pre-treatment showing promise in improving fiber radiation resistance. Mechanistic studies are relatively comprehensive, indicating significant development potential and suggesting that it may become the mainstream pre-treatment method for radiation-resistant fibers in the future.

## 4. Summary and Prospects

Currently, the development approaches for radiation-resistant optical fibers can be broadly categorized as follows:

(1) Fluorine doping: Fluorine-doped optical fibers represent one of the mainstream development approaches for radiation-resistant fibers. Although the underlying mechanisms and optimal doping concentrations are not yet fully understood, the current research indicates that fluorine doping can significantly enhance the radiation resistance of optical fibers.
(2) Metal element doping: Metal-element-doped optical fibers represent another mainstream development approach for radiation-resistant fibers. Among them, erbium-doped, ytterbium-doped, and erbium/ytterbium co-doped fibers have been well developed and are widely used in deep space exploration, ground communications, and other fields. It is worth noting that cerium doping has also been reported to improve the radiation resistance of certain types of optical fibers, showing some development potential. However, germanium doping does not effectively enhance the radiation resistance of optical fibers and is only applicable to certain specialized areas. Therefore, germanium-doped fibers may not be the mainstream direction for future radiation-resistant fiber development.
(3) Nitrogen doping: There are relatively few reports on nitrogen-doped optical fibers currently, but limited studies suggest that nitrogen doping can effectively improve the radiation resistance of optical fibers. Particularly, fiber Bragg gratings made from nitrogen-doped fibers have shown excellent performance in radiation environments.
(4) Various pre-treatment methods: Pre-treatment methods for radiation-resistant optical fibers mainly include gas loading, pre-irradiation, and pre-dehydration. Gas loading pre-treatment has matured and can effectively enhance the radiation resistance of optical fibers, with relatively well-understood mechanisms.
(5) Aluminum-doped and phosphorus-doped fibers: These types of fibers exhibit good sensitivity to radiation-induced attenuation (RIA), making them suitable for manufacturing high-performance radiation dosimeters. Although this characteristic does not meet the requirements for excellent radiation resistance, as needed for communication fibers, leveraging this property enables the production of excellent radiation dosimeters. It is worth noting that radiation dosimeters made from phosphorus-doped fibers not only possess many desirable characteristics but have also been demonstrated to have the potential for recovery and reuse. This indicates that phosphorus-doped fiber radiation dosimeters have a longer lifespan and lower maintenance costs, showcasing significant development potential.

Prospects: The development of radiation-resistant optical fibers has spanned several decades, during which researchers in relevant fields have developed various manufacturing processes for these fibers. Among them, erbium-doped fibers (EDFs) have been widely applied, with relatively well-understood mechanisms. Additionally, there are reports indicating that certain specialized manufacturing processes can further enhance the radiation resistance of EDFs, demonstrating their potential for further development. It is worth noting that erbium/ytterbium co-doped fibers (EYDFs) have also exhibited excellent radiation resistance. Compared to traditional YDFs, EYDFs demonstrate more stable communication performance in harsh radiation environments, such as outer space. However, the mechanisms of EYDFs are not yet fully understood, and further research is needed to achieve their commercial application.

Furthermore, fluorine-doped fibers have proven to be highly radiation-resistant and have already achieved some commercial applications. However, the underlying mechanisms of fluorine doping remain to be fully explored. The optimal doping concentration and specific mechanisms of fluorine’s action require further investigation and discussion. With an improved understanding of fluorine-doped fiber mechanisms, fluorine-doped fibers are expected to find broader commercial applications in the future.

In addition to the aforementioned reports, pre-treatment methods are also extensively reported in the manufacturing of radiation-resistant optical fibers. Several pre-treatment methods have been developed, with gas loading pre-treatment being the most significant. However, this method still has certain limitations. For example, deuterium loading pre-treatment may increase the loss of hydroxyl groups in optical fibers. While the presence of hydroxyl groups can reduce the initial concentration of precursors leading to color center formation, this phenomenon does not contribute to improving the radiation resistance of optical fibers and requires further improvement in subsequent research.

Other methods, such as the cerium doping, germanium doping, and nitrogen doping of fibers, started relatively later compared to traditional EDFs, YDFs, and fluorine-doped fibers. They have not yet matured in research, and some have only been proven to be applicable to certain specialized fields (e.g., germanium-doped fibers). Although cerium-doped fibers and nitrogen-doped fibers have been reported to possess excellent radiation resistance, research in these areas is still limited, and significant strides need to be made before achieving mature commercial applications.

## Figures and Tables

**Table 1:** Three types of fibers used by S. Girard et al. [110].

Fiber	[Er^3+^]aa	[Al_2_O_3_]
#1	~290 ppm	10 wt.%
#2	~240 ppm	8 wt.%
#2	~290 ppm	7 wt.%

**Table 2:** Changes in fiber RIA under measurements of two different energy levels of proton radiation.

Fiber #1	χ (dBm^−1^Gy^−1^, 50 Mev)	χ (dBm^−1^Gy^−1^, 105 Mev)
3 mW	-	1.1 × 10^−2^
23 mW	-	1.1 × 10^−2^
54 mW	-	1.1 × 10^−2^
**Fiber #2**	**χ (dBm** **^−^** **^1^** **Gy** **^−^** **^1^** **, 50 Mev)**	**χ (dBm** **^−^** **^1^** **Gy** **^−^** **^1^** **, 105 Mev)**
3 mW	1.1 × 10^−2^	1.6 × 10^−2^
23 mW	1.1 × 10^−2^	1.6 × 10^−2^
54 mW	1.1 × 10^−2^	1.6 × 10^−2^
**Fiber #3**	**χ (dBm** **^−^** **^1^** **Gy** **^−^** **^1^** **, 50 Mev)**	**χ (dBm** **^−^** **^1^** **Gy** **^−^** **^1^** **, 105 Mev)**
3 mW	9.6 × 10^−3^	1.1 × 10^−2^
23 mW	9.4 × 10^−3^	1.1 × 10^−2^
54 mW	8.8 × 10^−3^	1.2 × 10^−2^

## References

[1] Wang J., Zhou Y., Xu L., Jiang L., Wang L. (2023). Magnetic kirigami by laser cutting. Acta Mech. Solida. Sin..

[2] Krüger J., Bonse J. (2023). Special Issue “Advanced Pulse Laser Machining Technology”. Materials.

[3] Lin O., Xiao G., Liu S., Zhou Y., Liu Z., Huang J., Yin K. (2023). Rapid multiscale surface texture manufacturing process using hybrid laser belt machining. J. Mater. Process. Technol..

[4] Dodd N., Ballantyne E., Heron G., Goodall R. (2023). Multi-layer laser cutting of electrical steel sheets applied to electric machine laminations. PLoS ONE.

[5] Khan F.N. (2023). Data perspectives in AI-assisted fiber-optic communication networks. IEEE Netw..

[6] Khan F.N. (2022). Machine learning-enabled intelligent fiber-optic communications: Major obstacles and the way forward. IEEE Commun. Mag..

[7] Chagnon M., Morita I. (2022). Series Editorial: Optical Communications and Networks. IEEE Commun. Mag..

[8] Wang X., Chen G., Zhang K., Li R.A., Jiang Z., Zhou H., Gan J., He M. (2023). Self-healing multimodal flexible optoelectronic fiber sensors. Chem. Mater..

[9] Ran Y., Xu Z., Chen M., Wang W., Wu Y., Cai J., Long J., Chen Z.S., Zhang D., Guan B.O. (2022). Fiber-optic theranostics (FOT): Interstitial fiber-optic needles for cancer sensing and therapy. Adv. Sci..

[10] Kim K.J., Culp J.T., Wuenschell J., Shugayev R.A., Ohodnicki P.R., Sekizkardes A.K. (2023). Sorption-Induced Fiber Optic Plasmonic Gas Sensing via Small Grazing Angle of Incidence. Adv. Mater..

[11] Wang J., Man H.Y., Meng C., Liu P., Li S., Kwok H.S., Zi Y. (2021). A fully self-powered, natural-light-enabled fiber-optic vibration sensing solution. Susmat.

[12] Zhang X., Yang J., Qiang Y., Zhang Z., Wang X., Xue Y., Yuan Y., Wang Y., Qin Y. (2023). High spatial resolution internal stress testing and analysis of fiber optic winding structure using BOTDA. J. Light. Technol..

[13] Bao X. (2023). Prospects on ultrasound measurement techniques with optical fibers. Meas. Sci. Technol..

[14] Rong Q., Wang Y., Shao Z., Qiao X. (2018). Large diameter fiber-optics tweezers for escherichia coli bacteria manipulation. IEEE J. Sel. Top. Quantum. Electron..

[15] Collard L., Pisano F., Zheng D., Balena A., Kashif M.F., Pisanello M., D’Orazio A., De La Prida L.M., Ciracì C. (2022). Holographic Manipulation of Nanostructured Fiber Optics Enables Spatially-Resolved, Reconfigurable Optical Control of Plasmonic Local Field Enhancement and SERS. Small.

[16] López-Lapeña O., Polo-Cantero J. (2022). Time-Division Multiplexing for Power and Data Transmission on Optical Fibers. IEEE Internet. Things J..

[17] Bu F., Luo H., Ma S., Li X., Ruby R., Han G. (2023). AUV-Aided Optical—Acoustic Hybrid Data Collection Based on Deep Reinforcement Learning. Sensors.

[18] Akinci A., Bowden M.D., Cheeseman M.C., Knowles S.L., Meister D.C., Pecak S.N., Potter K.S. (2009). The effect of high dose rate transient gamma radiation on high-energy optical fibers. Optical Technologies for Arming, Safing, Fuzing, and Firing V.

[19] Sporea D., Sporea A., O’Keeffe S., Mccarthy D., Lewis E. (2012). Optical Fibers and Optical Fiber Sensors Used in Radiation Monitoring.

[20] Butov O.V., Chamorovskiy Y.K., Bazakutsa A.P., Fedorov A.N., Igor’A S. Optical fiber sensor for deformation monitoring of fuel channels in industrial nuclear reactors. Proceedings of the 26th International Conference on Optical Fiber Sensors.

[21] Cheymol G., Maurin L., Remy L., Arounassalame V., Maskrot H., Rougeault S., Dauvois V., Le Tutour P., Huot N., Ouerdane Y. (2020). Irradiation tests of optical fibers and cables devoted to corium monitoring in case of a severe accident in a nuclear power plant. IEEE Trans. Nucl. Sci..

[22] Hyer H.C., Giuliano D.R., Petrie C.M. (2023). Toward local core outlet temperature monitoring in gas-cooled nuclear reactors using distributed fiber-optic temperature sensors. Appl. Therm. Eng..

[23] Pouyat D., Couston L., Noire M.H., Davin T., Delage J., Bouzon C., Marty P. Real time analysis by in line spectrophotometry using optical fibre: Application to nuclear fuel reprocessing solutions. Proceedings of the RECOD 98.5. International Conference on Recycling, Conditioning and Disposal.

[24] Matsunaga T., Okochi M. (1995). TiO_2_-mediated photochemical disinfection of Escherichia coli using optical fibers. Environ. Sci. Technol..

[25] Westerhoff P., Zhao Z., Shapiro N. Nanoparticle enabled optical fibers to side-emit germicidal ultraviolet light for biofilm control in water. Proceedings of the Novel Optical Materials and Applications.

[26] Yin H., Li Y., Xing F., Wu B., Zhou Z., Zhang W. Hybrid acoustic, wireless optical and fiber-optic underwater cellular mobile communication networks. Proceedings of the 2018 IEEE 18th International Conference on Communication Technology (ICCT).

[27] Quattrocchi G., Berri P.C., Dalla M.V., Maggiore P. (2021). Optical fibers applied to aerospace systems prognostics: Design and development of new FBG-based vibration sensors. IOP Conf. Ser. Mater. Sci. Eng..

[28] Ladaci A., Girard S., Mescia L., Robin T., Laurent A., Cadier B., Boutillier M., Morana A., Di Francesca D., Ouerdane Y. (2018). X-rays, γ-rays, electrons and protons radiation-induced changes on the lifetimes of Er^3+^ and Yb^3+^ ions in silica-based optical fibers. J. Lumin..

[29] Girard S., Keurinck J., Ouerdane Y., Meunier J.P., Boukenter A., Derep J.L., Azais B., Charre P., Vie M. (2004). Pulsed X-ray and/spl gamma/rays irradiation effects on polarization-maintaining optical fibers. IEEE Trans. Nucl. Sci..

[30] Girard S., Baggio J., Bisutti J. (2006). 14-MeV Neutron, γ-Ray, and Pulsed X-Ray Radiation-Induced Effects on Multimode Silica-Based Optical Fibers. IEEE Trans. Nucl. Sci..

[31] Prajzler V., Masopoustova K., Sarsounova Z. (2022). Gamma radiation effects on plastic optical fibers. Opt. Fiber Technol..

[32] Pacchioni G., Skuja L., Griscom D.L. (2000). Defects in SiO_2_ and Related Dielectrics: Science and Technology.

[33] Griscom D.L., Levy P.W. (1985). Nature of defects and defect generation in optical glasses. Radiation Effects on Optical Materials.

[34] Wang B., Cao C., Xing Y.B., Chen G., Dai N.L., Li H.Q., Peng J.G., Li J.Y. (2021). Research Status on Radiation Performance and Radiation Resistance Technology of Rare-Earth-Doped Fibers. Laser Optoelectron. Prog..

[35] Wang Z.L., Cheng L.F. (2017). Irradiation Effects and Irradiation Resistance Modification of Glasses. Mater. Rep..

[36] Nicoletta C., Eubanks A. (1972). Effect of simulated space radiation on selected optical materials. Appl. Opt..

[37] Xing R.X. (2016). Study on Irradiance Resistance of Erbium-Doped Silicate Glass and Its Fiber. Master’s Thesis.

[38] Griscom D.L. (1989). Self-trapped holes in amorphous silicon dioxide. Phys. Rev. B.

[39] Johnston A.H. Radiation damage of electronic and optoelectronic devices in space. Proceedings of the 4th International Workshop on Radiation Effects on Semiconductor Devices for Space Application.

[40] Holcomb D.E., Miller D.W. (1993). A comparison of neutron and gamma damage effects on silica glass in a nuclear reactor radiation environment. J. Nucl. Mater..

[41] Shao C.Y., Yu C.L., Hu L.L. (2020). Radiation-Resistant Active Fibers for Space Applications. Chin. J. Lasers.

[42] Luo W.Y. (2014). Study on the Formation Mechanism of Radiation-Induced Defects in Quartz Fiber Materials. Doctoral Thesis.

[43] Imai H., Hirashima H. (1994). Intrinsic-and extrinsic-defect formation in silica glasses by radiation. J. Non-Cryst. Solids.

[44] Suratwala T., Steele W., Feit M., Moreno K., Stadermann M., Fair J., Chen K., Nikroo A., Youngblood K., Wu K. (2012). Polishing and local planarization of plastic spherical capsules using tumble finishing. Appl. Surf. Sci..

[45] Chai Y., Zhu M., Xing H., Wang H., Cui Y., Shao J. (2016). Multilayer deformation planarization by substrate pit suturing. Opt. Lett..

[46] Cheng J., Yang H., Liu Q., Zhao L., Liu Z., Liu H., Wang T., Xiao Y., Hu K., Chen M. (2019). Characterization of manufacturing-induced surface scratches and their effect on laser damage resistance performance of diamond fly-cut KDP crystal. Results Phys..

[47] Esqueda-Barrón Y., Herrera M., Camacho-López S. (2018). ZnO synthesized in air by fs laser irradiation on metallic Zn thin films. Appl. Surf. Sci..

[48] Sun L., Liu H., Huang J., Ye X., Xia H., Li Q., Jiang X., Wu W., Yang L., Zheng W. (2016). Reaction ion etching process for improving laser damage resistance of fused silica optical surface. Opt. Express.

[49] Li Y., Yan H., Yang K., Yao C., Wang Z., Zou X., Yan C., Yuan X., Ju X., Yang L. (2017). Surface molecular structure defects and laser-induced damage threshold of fused silica during a manufacturing process. Sci. Rep..

[50] Sun L., Shao T., Zhou X., Li W., Li F., Ye X., Huang J., Chen S., Li B., Yang L. (2021). Understanding the effect of HF-based wet shallow etching on optical performance of reactive-ion-etched fused silica optics. RSC Adv..

[51] Girard S., Kuhnhenn J., Gusarov A., Brichard B., Van Uffelen M., Ouerdane Y., Boukenter A., Marcandella C. (2013). Radiation effects on silica-based optical fibers: Recent advances and future challenges. IEEE Trans. Nucl. Sci..

[52] Williams R.T. (1985). Nature of defects and defect generation in optical crystals. Radiation Effects on Optical Materials.

[53] Morita Y., Kawakami W. (1989). Dose rate effect on radiation induced attenuation of pure silica core optical fibres. IEEE Trans. Nucl. Sci..

[54] Griscom D., Gingerich M., Friebele E. (1993). Radiation-induced defects in glasses: Origin of power-law dependence of concentration on dose. Phys. Rev. Lett..

[55] Borgermans P., Brichard B. (2002). Kinetic models and spectral dependencies of the radiation-induced attenuation in pure silica fibers. IEEE Trans. Nucl. Sci..

[56] Rizzolo S., Boukenter A., Allanche T., Périsse J., Bouwmans G., El Hamzaoui H., Bigot L., Ouerdane Y., Cannas M., Bouazaoui M. (2016). Optical frequency domain reflectometer distributed sensing using microstructured pure silica optical fibers under radiations. IEEE Trans. Nucl. Sci..

[57] Di Francesca D., Girard S., Agnello S., Alessi A., Marcandella C., Paillet P., Richard N., Boukenter A., Ouerdane Y., Gelardi F.M. (2016). Radiation response of ce-codoped germanosilicate and phosphosilicate optical fibers. IEEE Trans. Nucl. Sci..

[58] Weber W.J., Ewing R.C., Angell C.A., Arnold G.W., Cormack A.N., Delaye J.M., Griscom D.L., Hobbs L.W., Navrotsky A., Price D.L. (1997). Radiation effects in glasses used for immobilization of high-level waste and plutonium disposition. J. Mater. Res..

[59] Nürnberg F., Kühn B., Langner A., Altwein M., Schötz G., Takke R., Thomas S., Vydra J. (2015). Bulk damage and absorption in fused silica due to high-power laser applications. Laser-Induced Damage in Optical Materials.

[60] Kordas G., Camara B., Oel H.J. (1982). Electron spin resonance studies of radiation damage in silicate glasses. J. Non-Cryst. Solids.

[61] Du J.S., Wu J.H., Zhao L.L., Song L.X. (2012). Coloration of Glasses Induced by Space Ionizing Radiation. J. Inorg. Mater..

[62] Prabhu N.S., Somashekarappa H.M., Sayyed M., Osman H., Alamri S., Khandaker M.U., Kamath S.D. (2021). Structural and optical modifications in the BaO-ZnO-LiF-B_2_O_3_-Yb_2_O_3_ glass system after γ-irradiation. Materials.

[63] Griscom D., Gingerich M., Friebele E. (1994). Model for the dose, dose-rate and temperature dependence of radiation-induced loss in optical fibers. IEEE Trans. Nucl. Sci..

[64] Henschel H., Koehn O., Schmidt H.U. (1991). Influence of dose rate on radiation-induced loss in optical fibers. Optical Systems in Adverse Environments.

[65] Friebele E.J., Askins C.G., Gingerich M.E. (1984). Effect of low dose rate irradiation on doped silica core optical fibers. Appl. Opt..

[66] Ramsey A., Tighe W., Bartolick J., Morgan P. (1997). Radiation effects on heated optical fibers. Rev. Sci. Instrum..

[67] Girard S., Marcandella C., Morana A., Perisse J., Di Francesca D., Paillet P., Macé J.R., Boukenter A., Léon M., Gaillardin M. (2013). Combined high dose and temperature radiation effects on multimode silica-based optical fibers. IEEE Trans. Nucl. Sci..

[68] Henschel H., Kohn O. (2000). Regeneration of irradiated optical fibres by photobleaching. IEEE Trans. Nucl. Sci..

[69] Borgermans P., Noel M. Multiple wavelength analysis of radiation induced attenuation on optical fibres: A novel approach in fibre optic dosimetry. Proceedings of the IMTC/98 Conference Proceedings. IEEE Instrumentation and Measurement Technology Conference. Where Instrumentation is Going.

[70] Regnier E., Flammer I., Girard S., Gooijer F., Achten F., Kuyt G. (2007). Low-dose radiation-induced attenuation at infrared wavelengths for P-doped, Ge-doped and pure silica-core optical fibres. IEEE Trans. Nucl. Sci..

[71] Girard S., Boukenter A., Ouerdane Y., Meunier J.P., Keurinck J. (2003). Properties of phosphorus-related defects induced by γ-rays and pulsed X-ray irradiation in germanosilicate optical fibers. J. Non-Cryst. Solids.

[72] Brichard B., Agnello S., Nuccio L., Dusseau L. (2008). Comparison Between Point Defect Generation by γ-rays in Bulk and Fibre Samples of High Purity Amorphous SiO_2_. IEEE Trans. Nucl. Sci..

[73] Girard S., Marcandella C., Alessi A., Boukenter A., Ouerdane Y., Richard N., Paillet P., Gaillardin M., Raine M. (2012). Transient radiation responses of optical fibers: Influence of MCVD process parameters. IEEE Trans. Nucl. Sci..

[74] Origlio G., Cannas M., Girard S., Boscaino R., Boukenter A., Ouerdane Y. (2009). Influence of the drawing process on the defect generation in multistep-index germanium-doped optical fibers. Opt. Lett..

[75] Kuhnhenn J., Henschel H., Weinand U. Influence of coating material, cladding thickness, and core material on the radiation sensitivity of pure silica core step-index fibers. Proceedings of the 2005 8th European Conference on Radiation and Its Effects on Components and Systems.

[76] Vecchi G.L., Di Francesca D., Sabatier C., Girard S., Alessi A., Guttilla A., Robin T., Kadi Y., Brugger M. (2020). Infrared radiation induced attenuation of radiation sensitive optical fibers: Influence of temperature and modal propagation. Opt. Fiber Technol..

[77] Giacomazzi L., Martin-Samos L., Boukenter A., Ouerdane Y., Girard S., Alessi A., De Gironcoli S., Richard N. (2017). Photoactivated processes in optical fibers: Generation and conversion mechanisms of twofold coordinated Si and Ge atoms. Nanotechnology.

[78] Uchino T., Yoko T. (2006). Density functional theory of structural transformations of oxygen-deficient centers in amorphous silica during hole trapping: Structure and formation mechanism of the Eγ′ center. Phys. Rev. B.

[79] Uchino T., Takahashi M., Yoko T. (2001). Structure and generation mechanism of the peroxy-radical defect in amorphous silica. Phys. Rev. Lett..

[80] Uchino T., Takahashi M., Yoko T. (2002). Formation and decay mechanisms of electron–hole pairs in amorphous SiO_2_. Appl. Phys. Lett..

[81] Devine R.A.B., Duraud J.P., Dooryhee E. (2000). Structure and Imperfections in Amorphous and Crystalline Silicon Dioxide.

[82] Kameyama A., Yokotani A., Kurosawa K. (2002). Generation and erasure of second-order optical nonlinearities in thermally poled silica glasses by control of point defects. JOSA B.

[83] Girard S., Alessi A., Richard N., Martin-Samos L., De Michele V., Giacomazzi L., Agnello S., Di Francesca D., Morana A., Winkler B. (2019). Overview of radiation induced point defects in silica-based optical fibers. Rev. Phys..

[84] O’Keeffe S., Lewis E., Santhanam A., Rolland J.P. Variable sensitivity online optical fibre radiation dosimeter. Proceedings of the SENSORS.

[85] Chapalo I., Gusarov A., Ioannou A., Pospori A., Chah K., Nan Y.G., Kalli K., Mégret P. (2022). Online gamma radiation monitoring using few-mode polymer CYTOP fiber Bragg gratings. Sensors.

[86] Golant K.M., Pacchioni G., Skuja L., Griscom D.L. (2000). Bulk silicas prepared by low pressure plasma CVD: Formation of structure and point defects. Defects in SiO_2_ and Related Dielectrics: Science and Technology.

[87] Piao F., Oldham W.G., Haller E.E. (2000). Ultraviolet-induced densification of fused silica. J. Appl. Phys..

[88] Blanco S.G., Glidle A., Cooper J.M., De La Rue R.M., Jacqueline A.S., Poumellec B., Aitchison J.S. Characterization of the densification induced by electron-beam irradiation of ge-doped silica for the fabrication of integrated optical circuits. Proceedings of the 2002 IEEE/LEOS Workshop on Fibre and Optical Passive Components.

[89] Salleo A., Taylor S.T., Martin M.C., Panero W.R., Jeanloz R., Sands T., Génin F.Y. (2003). Laser-driven formation of a high-pressure phase in amorphous silica. Nat. Mater..

[90] Buscarino G., Agnello S., Gelardi F. (2009). Structural modifications induced by electron irradiation in SiO2 glass: Local densification measurements. Europhys. Lett..

[91] Vigouroux J., Duraud J., Le Moel A., Le Gressus C., Griscom D. (1985). Electron trapping in amorphous SiO_2_ studied by charge buildup under electron bombardment. J. Appl. Phys..

[92] Wang R., Tai N., Saito K., Ikushima A. (2005). Fluorine-doping concentration and fictive temperature dependence of self-trapped holes in SiO_2_ glasses. J. Appl. Phys..

[93] Sanada K., Kakuta T. (1993). Method of Manufacturing Radiation-Resistant Optical Fiber. U.S. Patent.

[94] Sanada K., Shamoto N., Inada K. (1994). Radiation resistance of fluorine-doped silica-core fibers. J. Non-Cryst. Solids.

[95] Wijnands T., Aikawa K., Kuhnhenn J., Ricci D., Weinand U. (2011). Radiation tolerant optical fibers: From sample testing to large series production. J. Light. Technol..

[96] Stajanca P., Krebber K. Towards on-line radiation monitoring with perfluorinated polymer optical fibers. Proceedings of the 2017 25th Optical Fiber Sensors Conference (OFS).

[97] Lethien C., Loyez C., Vilcot J.P., Rolland N. A multi-hop UWB radio over polymer fibre system for 60-GHz hybrid network. Proceedings of the European Workshop on Photonic Solutions for Wireless, Access, and in House Networks.

[98] Chapalo I., Gusarov A., Kinet D., Chah K., Nan Y.G., Mégret P. (2022). Postirradiation transmission characteristics of CYTOP fiber exposed by gamma radiation. IEEE Trans. Nucl. Sci..

[99] Chapalo I., Guasarov A., Kinet D., Chah K., Nan Y.G., Megret P. (2021). Long-term Transmission Characteristics of CYTOP Fiber Exposed to Gamma Radiation. EPJ Web Conf..

[100] Wijnands T., De Jonge L.K., Kuhnhenn J., Hoeffgen S.K., Weinand U. (2008). Optical absorption in commercial single mode optical fibers in a high energy physics radiation field. IEEE Trans. Nucl. Sci..

[101] Pal A., Dhar A., Ghosh A., Sen R., Hooda B., Rastogi V., Ams M., Fabian M., Sun T., Grattan K. (2017). Sensors for harsh environment: Radiation resistant FBG sensor system. J. Light. Technol..

[102] Stajanca P., Krebber K. (2017). Radiation-induced attenuation of perfluorinated polymer optical fibers for radiation monitoring. Sensors.

[103] Blanc J., Ricci D., Kuhnhenn J., Weinand U., Schumann O.J. (2017). Irradiation of radiation-tolerant single-mode optical fibers at cryogenic temperature. J. Light. Technol..

[104] Williams G.M., Putnam M.A., Askins C.G., Gingerich M.E., Friebele E.J. (1993). Radiation-induced coloring of erbium-doped optical fibers. Optical Materials Reliability and Testing: Benign and Adverse Environments.

[105] Takahara M., Yokoo T., Gomi H. Splice effects of Er-doped fiber in Er-doped fiber amplifiers. Proceedings of the ICCS’94.

[106] Fukuda C., Chigusa Y., Kashiwada T., Onishi M., Kanamori H., Okamoto S. (1994). γ-ray irradiation durability of erbium-doped fibres. Electron. Lett..

[107] Ladaci A., Girard S., Mescia L., Robin T., Laurent A., Cadier B., Boutillier M., Ouerdane Y., Boukenter A. (2017). Optimized radiation-hardened erbium doped fiber amplifiers for long space missions. J. Appl. Phys..

[108] Williams G., Putnam M., Askins C., Gingerich M., Friebele E. (1992). Radiation effects in erbium-doped optical fibres. Electron. Lett..

[109] Rose T.S., Gunn D., Valley G.C. (2001). Gamma and proton radiation effects in erbium-doped fiber amplifiers: Active and passive measurements. J. Light. Technol..

[110] Girard S., Tortech B., Regnier E., Van Uffelen M., Gusarov A., Ouerdane Y., Baggio J., Paillet P., Ferlet-Cavrois V., Boukenter A. (2007). Proton-and gamma-induced effects on erbium-doped optical fibers. IEEE Trans. Nucl. Sci..

[111] Gusarov A., Van Uffelen M., Hotoleanu M., Thienpont H., Berghmans F. (2009). Radiation sensitivity of EDFAs based on highly Er-doped fibers. J. Light. Technol..

[112] Li M., Ma J., Tan L., Zhou Y., Yu S., Yu J., Che C. (2009). Investigation of the irradiation effect on erbium-doped fiber amplifiers composed by different density erbium-doped fibers. Laser Phys..

[113] Thomas J., Myara M., Troussellier L., Burov E., Pastouret A., Boivin D., Mélin G., Gilard O., Sotom M., Signoret P. (2012). Radiation-resistant erbium-doped-nanoparticles optical fiber for space applications. Opt. Express.

[114] Griscom D.L. (2001). Fractal kinetics of radiation-induced point-defect formation and decay in amorphous insulators: Application to color centers in silica-based optical fibers. Phys. Rev. B.

[115] Williams G.M., Wright B.M., Mack W.D., Friebele E.J. (1999). Projecting the performance of erbium-doped fiber devices in a space radiation environment. Optical Fiber Reliability and Testing.

[116] Brichard B., Fernandez A.F., Ooms H., Berghmans F. Gamma dose rate effect in erbium-doped fibers for space gyroscopes. Proceedings of the 16th International Conference on Optical Fiber Sensors.

[117] Gilard O., Thomas J., Troussellier L., Myara M., Signoret P., Burov E., Sotom M. (2012). Theoretical explanation of enhanced low dose rate sensitivity in erbium-doped optical fibers. Appl. Opt..

[118] Girard S., Laurent A., Pinsard E., Raine M., Robin T., Cadier B., Di Francesca D., Paillet P., Gaillardin M., Duhamel O. (2014). Proton irradiation response of hole-assisted carbon coated erbium-doped fiber amplifiers. IEEE Trans. Nucl. Sci..

[119] Jiao Y., Yang Q., Guo M., Ma X., Shao C., Yu C., Hu L. (2021). Effect of the GeO_2_ content on the radiation resistance of Er^3+^-doped silica glasses and fibers. Opt. Mater. Express.

[120] Mady F., Benabdesselam M., Blanc W. (2010). Thermoluminescence characterization of traps involved in the photodarkening of ytterbium-doped silica fibers. Opt. Lett..

[121] Basu C. (2010). Photodarkening in Ytterbium Doped Silica Fibers under 488 nm CW Irradiation. Masters Thesis.

[122] D’Orazio A., De Sario M., Mescia L., Petruzzelli V., Prudenzano F. (2005). Refinement of Er^3+^-doped hole-assisted optical fiber amplifier. Opt. Express.

[123] Urquhart P. (1988). Review of rare earth doped fibre lasers and amplifiers. IEE Proc. J..

[124] Ma J., Li M., Tan L., Zhou Y., Yu S., Ran Q. (2009). Experimental investigation of radiation effect on erbium-ytterbium co-doped fiber amplifier for space optical communication in low-dose radiation environment. Opt. Express.

[125] Girard S., Ouerdane Y., Tortech B., Marcandella C., Robin T., Cadier B., Baggio J., Paillet P., Ferlet-Cavrois V., Boukenter A. (2009). Radiation effects on ytterbium-and ytterbium/erbium-doped double-clad optical fibers. IEEE Trans. Nucl. Sci..

[126] Dianov E., Golant K., Khrapko R., Tomashuk A. (1995). Nitrogen doped silica core fibres: A new type of radiation-resistant fibre. Electron. Lett..

[127] Bhatia V., Vengsarkar A.M. (1996). Optical fiber long-period grating sensors. Opt. Lett..

[128] Dianov E.M., Kurkov A.S., Medvedkov O.I., Vasiliev S.A. Photoinduced long-period fiber grating as a promising sensor element. Proceedings of the 10th European Conference on Solid-State Transducers.

[129] Ferdinand P., Magne S., Marty V., Rougeault S., Bernage P., Douay M., Fertein E., Lahoreau F., Niay P., Bayon J.F. (1994). Optical fibre Bragg grating sensors for structure monitoring within th nuclear power plants. Optical Fibre Sensing and Systems in Nuclear Environments.

[130] Liang L., Jiang D.S., Zhou X.F., Chen D.X. (2003). Applications of Fiber Bragg Grating Sensors in Civil Engineering. J. Luoyang Instit. Technol..

[131] Vasiliev S.A., Dianov E.M., Golant K.M., Medvedkov O.I., Tomashuk A.L., Karpov V.I., Grekov M.V., Kurkov A.S., Leconte B., Niay P. Performance of Bragg and long-period gratings written in N- and Ge-doped silica fibers under/spl gamma/-radiation. Proceedings of the RADECS 97. Fourth European Conference on Radiation and its Effects on Components and Systems.

[132] Girard S., Keurinck J., Boukenter A., Meunier J.P., Ouerdane Y., Azaıs B., Charre P., Vié M. (2004). Gamma-rays and pulsed X-ray radiation responses of nitrogen-, germanium-doped and pure silica core optical fibers. Nucl. Instrum. Meth. Phys. Res. B.

[133] Skuja L., Trukhin A., Plaudis A. (1984). Luminescence in germanium-doped glassy SiO_2_. Phys. Status Solidi A.

[134] Trukhin A., Poumellec B. (2004). Energy transport in silica to oxygen-deficient luminescence centers. Comparison with other luminescence centers in silica and α-quartz. Solid State Commun..

[135] Trukhin A., Boukenter A., Ouerdane Y., Girard S. (2011). γ-ray induced GeODC (II) centers in germanium doped α-quartz crystal. J. Non-Cryst. Solids.

[136] Kakuta T., Shikama T., Narui M., Sagawa T. (1998). Behavior of optical fibers under heavy irradiation. Fusion Eng. Des..

[137] Shikama T., Kakuta T., Shamoto N., Narui M., Sagawa T. (2000). Behavior of developed radiation-resistant silica-core optical fibers under fission reactor irradiation. Fusion Eng. Des..

[138] Benabdesselam M., Mady F., Girard S., Mebrouk Y., Duchez J.B., Gaillardin M., Paillet P. (2013). Performance of Ge-doped optical fiber as a thermoluminescent dosimeter. IEEE Trans. Nucl. Sci..

[139] Eronyan M., Devetyarov D., Reutskiy A., Untilov A., Aksarin S., Meshkovskiy I., Bisyarin M., Pechenkin A. (2021). MCVD method for manufacturing polarization-maintaining and radiation resistant optical fiber with germanosilicate elliptical core. Mater. Lett..

[140] Zhou P., Zhang R., Zhao H., Liu Y.H. (2023). Effect of CeO_2_ Content on Irradiation Resistance of Gallate Glass. Mater. Rep..

[141] Zu C.K., Chen J., Zhao H.F., Liu Y.H., Han B., Wang H.X. (2009). Effect of Rare Earth Ions(Gd^3+^,Ce^3+^/Ce^4+^, Eu^3+^) on Luminescence of Tb^3+^ Doped Silicate Glasses. Glass Enamel.

[142] Kadono K., Itakura N., Akai T., Yamashita M., Yazawa T. (2009). Effect of additive ions on the optical density and stability of the color centers induced by X-ray irradiation in soda-lime silicate glass. Nucl. Instrum. Meth. Phys. Res. B.

[143] Engholm M., Norin L. Ytterbium-doped fibers co-doped with cerium: Next generation of fibers for high power fiber lasers?. Proceedings of the Fiber Lasers VII: Technology, Systems, and Applications.

[144] Koumvakalis N. (1980). Defects in crystalline SiO_2_: Optical absorption of the aluminum-associated hole center. J. Appl. Phys..

[145] Trukhin A., Teteris J., Fedotov A., Griscom D., Buscarino G. (2009). Photosensitivity of SiO_2_–Al and SiO_2_–Na glasses under ArF (193 nm) laser. J. Non-Cryst. Solids.

[146] Agnello S., Boizot B. (2003). Transient visible-UV absorption in beta irradiated silica. J. Non-Cryst. Solids.

[147] Alessi A., Guttilla A., Girard S., Agnello S., Cannas M., Robin T., Boukenter A., Ouerdane Y. (2019). Radiation effects on aluminosilicate optical fibers: Spectral investigations from the ultraviolet to near-infrared domains. Phys. Status Solidi A.

[148] Alessi A., Guttilla A., Agnello S., Sabatier C., Robin T., Barnini A., Di Francesca D., Vecchi G.L., Cannas M., Boukenter A. (2021). Near-IR Radiation-Induced Attenuation of Aluminosilicate Optical Fibers. Phys. Status Solidi A.

[149] West R., Buker H., Friebele E., Henschel H., Lyons P. (1994). The use of optical time domain reflectometers to measure radiation-induced losses in optical fibers. J. Light. Technol..

[150] Henschel H., Koerfer M., Kuhnhenn J., Weinand U., Wulf F. Fibre optic sensor solutions for particle accelerators. Proceedings of the 17th International Conference on Optical Fibre Sensors.

[151] Berghmans F., Brichard B., Fernandez A.F., Gusarov A., Uffelen M.V., Girard S., Bock W.J., Gannot I., Tanev S. (2008). An introduction to radiation effects on optical components and fiber optic sensors. Optical Waveguide Sensing and Imaging.

[152] Faustov A., Gussarov A., Wuilpart M., Fotiadi A.A., Liokumovich L.B., Kotov O.I., Zolotovskiy I.O., Tomashuk A.L., Deschoutheete T., Mégret P. (2012). Distributed optical fibre temperature measurements in a low dose rate radiation environment based on Rayleigh backscattering. Optical Sensing and Detection II.

[153] Faustov A., Gusarov A., Wuilpart M., Fotiadi A., Liokumovich L., Zolotovskiy I., Tomashuk A., De Schoutheete T., Megret P. (2013). Comparison of gamma-radiation induced attenuation in Al-doped, P-doped and Ge-doped fibres for dosimetry. IEEE Trans. Nucl. Sci..

[154] Di Francesca D., Girard S., Agnello S., Alessi A., Marcandella C., Paillet P., Ouerdane Y., Kadi Y., Brugger M., Boukenter A. (2019). Combined Temperature Radiation Effects and Influence of Drawing Conditions on Phosphorous-Doped Optical Fibers. Phys. Status Solidi A.

[155] Di Francesca D., Vecchi G.L., Girard S., Morana A., Reghioua I., Alessi A., Hoehr C., Robin T., Kadi Y., Brugger M. (2019). Qualification and calibration of single-mode phosphosilicate optical fiber for dosimetry at CERN. J. Light. Technol..

[156] Vecchi G.L., Di Francesca D., Kadi Y., Ricci D., Brugger M., Campanella C., Alessi A., Ouerdane Y., Girard S. (2020). In-situ regeneration of P-doped optical fiber dosimeter. Opt. Lett..

[157] Girard S., Ouerdane Y., Bouazaoui M., Marcandella C., Boukenter A., Bigot L., Kudlinski A. (2011). Transient radiation-induced effects on solid core microstructured optical fibers. Opt. Express.

[158] Girard S., Baggio J., Leray J.L. (2005). Radiation-induced effects in a new class of optical waveguides: The air-guiding photonic crystal fibers. IEEE Trans. Nucl. Sci..

[159] Nagasawa K., Hoshi Y., Ohki Y., Yahagi K. (1985). Improvement of radiation resistance of pure silica core fibers by hydrogen treatment. Jpn. J. Appl. Phys..

[160] Griscom D.L. (1995). Radiation hardening of pure-silica-core optical fibers by ultra-high-dose γ-ray pre-irradiation. J. Appl. Phys..

[161] Ito C., Naito H., Nishimura A., Ohba H., Wakaida I., Sugiyama A., Chatani K. (2014). Development of radiation-resistant optical fiber for application to observation and laser spectroscopy under high radiation dose. J. Nucl. Sci. Technol..

[162] Shao C., Jiao Y., Lou F., Wang M., Zhang L., Feng S., Wang S., Chen D., Yu C., Hu L. (2020). Enhanced radiation resistance of ytterbium-doped silica fiber by pretreating on a fiber preform. Opt. Mater. Express.

[163] Jiao Y., Yang Q., Zhu Y., Wang F., Zhang L., Wang M., Wang S., Shao C., Yu C., Hu L. (2022). Improved radiation resistance of an Er-doped silica fiber by a preform pretreatment method. Opt. Express.

